# Clinical and Genetic Characteristics of Two Cases With Developmental and Epileptic Encephalopathy 93 Caused by Novel ATP6V1A Mutations and Literature Review

**DOI:** 10.1155/2024/4678670

**Published:** 2024-08-30

**Authors:** Jian Ma, Hongwei Zhang, Yuqiang Lv, Min Gao, Zhongtao Gai, Yi Liu

**Affiliations:** ^1^ Pediatric Research Institute Children's Hospital Affiliated to Shandong University (Jinan Children's Hospital), Jinan, China; ^2^ Pediatric Neurology Department Children's Hospital Affiliated to Shandong University (Jinan Children's Hospital), Jinan, China; ^3^ Shandong Provincial Clinical Research Center for Children's Health and Disease, Jinan, China

**Keywords:** *ATP6V1A* gene, developmental and epileptic encephalopathy, epilepsy, heterozygous mutation, whole exome sequencing

## Abstract

Developmental and epileptic encephalopathy 93 (DEE93) is a new defined autosomal dominant neurologic disorder caused by heterozygous mutations in the *ATP6V1A* gene on chromosome 3q13. DEE93 is characterized by developmental delay, early-onset refractory seizures, hypotonia, and intellectual disability. So far, merely 31 cases caused by *ATP6V1A* gene mutation have been reported in literature worldwide, and early genetic detection is required for differential diagnosis. Here, we analyze the clinical and genetic features of two patients with two novel *ATP6V1A* mutations (c.1061G>T/p.(Trp354Leu) and c.746C>T/p.(Pro249Leu)) and expound the therapeutic schedule for epilepsy. We also review the reported mutations and genotypes associated with the disorder. Our study expands the clinical and genetic spectrum of *ATP6V1A* mutation-associated DEE93, which provides a basis for the diagnosis, treatment, and genetic counseling of the disorder.

## 1. Introduction

Developmental and epileptic encephalopathies (DEEs) are severe neurologic disorders with frequent seizure activity, developmental delay, intellectual disability, or cognitive deficits [1, 2]. Although the underlying causes of DEEs are complex, increasing evidence suggests that infant and childhood-onset epilepsies are associated with heredity factors [3]. To date, above 90 genes associated with DEEs have been identified, and majority of DEE-associated gene variants are sporadic de novo rather than inherited [4–6]. *ATP6V1A* variants were initially identified in three unrelated families with autosomal recessive cutis laxa type IID (ARCL2D; MIM:617403), which is a connective tissue disorder characterized by cutis laxa, facial dysmorphism, and hypotonia, as well as a history of neurological complications [7, 8]. In 2018, Fassio et al. reported four cases of seizures and developmental delay with monoallelic *ATP6V1A* variants, which was the first study to connect *ATP6V1A* with developmental and epileptic encephalopathy-93 (DEE93; MIM:618012) [9]. DEE93 is a variable phenotype ranging from rapidly progressive and early fatal encephalopathies to mild–moderate intellectual disability, with or without seizures [10, 11].

The *ATP6V1A* gene (MIM#607027) located on chromosome 3q13.3 consists of 15 exons, encoding one subunit of the vacuolar-type H(+)-ATPase (V-ATPase), a protein that acts as an ATP-dependent protein pump involved in the intracellular proton trafficking processes and acidification of intracellular organelles [12, 13]. V-ATPase complex is composed of two primary functional domains known as a catalytic cytosolic V1 domain and an H+-pumping, membrane-embedded V0 domain [8]. The V1 domain contains eight distinct subunits from A to H, through binding to and hydrolyzing ATP. The V0 domain consists of five subunits and is responsible for proton translocation across the membranes [14]. The V-ATPase is predominantly expressed in neurons where it plays additional and unique roles in neurotransmitter loading into synaptic vesicles and in regulating synaptic transmission [15, 16]. In contrast to the majority of V-ATPase subunits, V1A does not have any isoforms. Studies showed *ATP6V1A* dominant variants caused V-ATPase functional defects involving alterations of lysosomal homeostasis and dysfunction, which markedly affected neurite development and synaptic connectivity, uncovering that V-ATPase plays an important role in neuronal development [9, 10]. In addition, *ATP6V1A* recessive variants were shown to affect protein glycosylation in patients with cutis laxa [8].

Up to now, 31 patients with *ATP6V1A* mutations presenting epileptic encephalopathy have been reported [9–11, 17, 18]. Through whole exome sequencing (WES), we identified additional two de novo heterozygous mutations of c.1061G>T/p.(Trp354Leu) and c.746C>T/p.(Pro249Leu) in the *ATP6V1A* gene in two unrelated patients. We described clinical phenotypes of both patients and analyzed the features of phenotypes and genotypes by reviewing all published reports of 31 patients with *ATP6V1A* mutations. More importantly, we added treatment options, which will provide a basis for the diagnosis, treatment, and genetic counseling of the disorder.

## 2. Materials and Methods

### 2.1. Clinical Samples

This work was approved by the Medical Ethics Committee of Children's Hospital Affiliated to Shandong University. Clinical and laboratory examinations were performed on the patients and their parents after informed consent was obtained. The information of all patients was anonymized before submission. All clinical investigation has been conducted according to the principles expressed in the Declaration of Helsinki.

### 2.2. Whole Exome and Sanger Sequencing

Genomic DNA was extracted from the peripheral blood of patient and parents. Informed consent for genetic testing was obtained from participants. WES with the Human Exome Probes P039-Exome (MyGenostics, Beijing, China) on the Illumina NovaSeq5000 platform (Illumina, United States) was applied for the mutation screening of the patient. The obtained mean exome coverage was more than 95% (> 10× coverage; mean depth of over 100×). The quality controlled reads were mapped to the human reference genome (UCSC hg19, NCBI build 37). Read mapping, variant calling, and annotation were performed, as previously described. Detected variants, short indels, and single nucleotide variants (SNVs) were annotated and ranked by VaRank software. Sorting Intolerant from Tolerant (SIFT), PolyPhen-2, MutationTaster, and REVEL were used to evaluate the novel variants' pathogenicity.

*ATP6V1A* gene variants identified by WES were confirmed in the patients, and the pathogenic or likely pathogenic variants were checked in their parents with designed specific primers by the Sanger sequencing. The primer sets were designed using Primer Premier v5.0 software. PCR amplification was performed using AmpliTaq Gold® 360 DNA polymerase (Applied Biosystems). PCR products were further purified and sequenced using an ABI Prism 3700 automated sequencer (Applied Biosystems, Foster City, CA).

## 3. Results

### 3.1. Clinical Characteristics and Treatments of the Affected Individuals

#### 3.1.1. Patient 1

**Figure 1:**
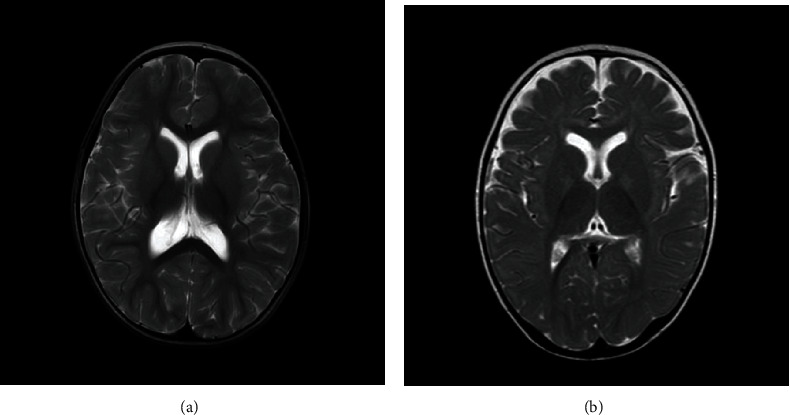
(a) Cranial MRI of Patient 1 showed hypomyelination and enlargement of lateral ventricles. (b) Cranial MRI of Patient 2 showed partial T2W signal in bilateral pallidus that was slightly higher, and some extracerebral fluid spaces were wider.

The patient, a girl aged 1 year and 5 months, was admitted to our hospital due to frequent seizures. She was born of natural birth without the history of asphyxia or hypoxia. Her parents were in a nonconsanguineous marriage, and there was no family history of epilepsy or other neurological and psychiatric conditions. Her birth weight was 2780 g, length 49 cm. No dysmorphisms were presented at birth. She had global developmental delay in infancy, first holding her head up at 7 months and sitting at 12 months. At the age of 17 months, her development still lags behind her peers. At the age of 1 year and 5 months, she developed seizures characterized by frequent nodding and hugging movements. Her seizures typically lasted for half a minute at a frequency of four or five times daily. A comprehensive physical examination found no evidence of abnormalities except for limb hypotonia. Laboratory tests, including serum amino acid quantification, blood routine test, blood biochemical test, blood lactic acid, blood ammonia concentration, thyroid function tests, and urine organic acid quantification, were all normal. No abnormality was observed in visual and auditory evoked potentials. A Gesell Developmental Scale (GDS) demonstrated severe developmental delay. Peabody exercise capacity assessment demonstrated GMQ66, FMQ55, and TMQ58. The result of karyotype analysis was 46XX. Her electroencephalogram (EEG) showed bilateral posterior head discharges and hypsarrhythmia during sleep period. Cranial MRI showed hypomyelination, broadening lateral ventricles (Figure 1(a)). Oral topiramate and intramuscular adrenocorticotrophic hormone (ACTH) therapy were first used but showed to be ineffective. Then, with the addition of levetiracetam and a ketogenic diet for 6 months, her seizure frequency reduced by 50% of baseline. Finally, she was added with vigabatrin (108.7 mg/kg/day), and her frequency of seizures decreased to 1–2 times/day. In addition, after undergoing short-term rehabilitation training, she was able to sit up unaided but was unable to walk. Her language did not contain meaningful words, and her interactions with others were poor.

#### 3.1.2. Patient 2

The patient, a 1-year-old girl, was admitted to our hospital because of developmental delay. She had a natural delivery without the history of asphyxia or hypoxia. Her parents were nonconsanguineous, and there was no family history of epilepsy or other neurological and psychiatric conditions. Her birth weight, length, and head circumference were not available. She only presented global developmental delay and hypotonia in infancy. So far, no seizures have been observed, and her EEG was normal. Cranial MRI showed slightly higher partial T2W signal in the bilateral pallidus and some wider extracerebral fluid spaces (Figure 1(b)). Laboratory tests, including serum amino acid quantification, blood routine test, blood biochemical test, blood lactic acid, blood ammonia concentration, thyroid function tests, and urine organic acid quantification, were all normal. No abnormality was observed in visual and auditory evoked potentials. Initially, her GDS demonstrated developmental delay, including language 39, adaptive 23, gross motor 33, fine motor 17, and personal-social 26. She was treated with L-carnitine, vitamin B12, calcium, and speech and exercise rehabilitation for 5 months. Her development has improved, with GDS showing the language 45, adaptive 40, gross motor 40, fine motor 35, and personal-social 45.

### 3.2. Identification of De Novo *ATP6V1A* Mutation

**Figure 2:**
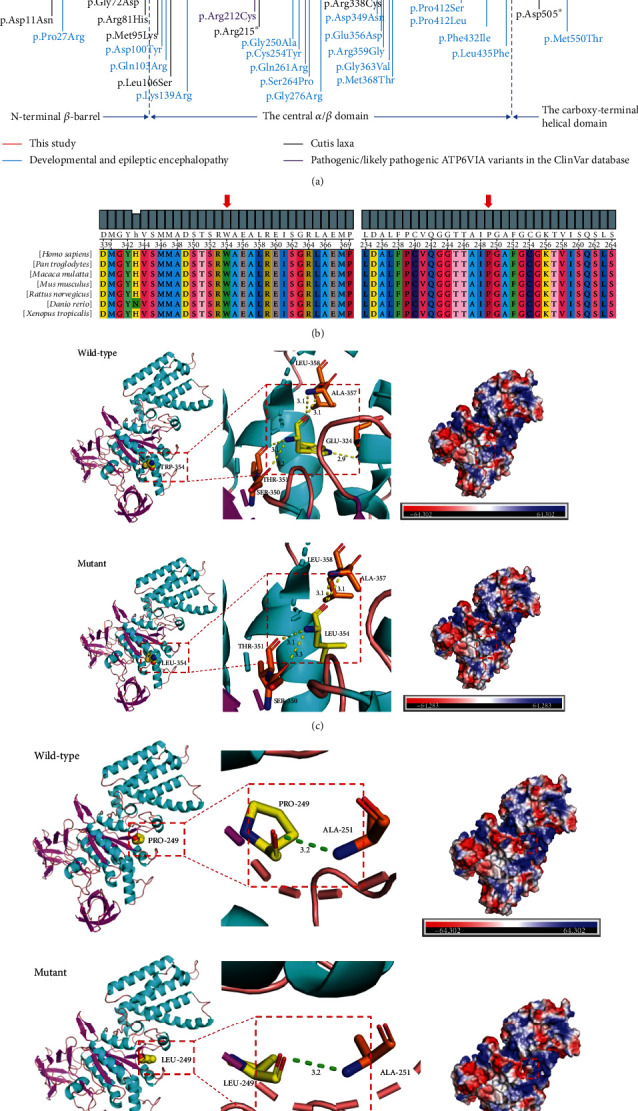
Identification of de novo *ATP6V1A* mutation. (a) Schematic representation of all (likely) pathogenic variants identified in *ATP6V1A*. Genomic structure of exons 2–14 of *ATP6V1A*. Variants associated with childhood epilepsy identified in this study were depicted in red, variants associated with DEE were depicted in blue, and variants associated with autosomal recessive cutis laxa were depicted in black. Purple annotation indicates mutations identified in the ClinVar database. (b) Multiple sequence alignments of ATP6V1A protein sequences showing that the “tryptophan” position 354 and the “proline” position 249 are highly conserved across different species. (c, d) Hydrogen bond changes of the *APT6V1A* mutants and changes of charge. Trp354 formed five hydrogen bonds with Glu324, Ser350, Thr351, Ala357, and Leu358, while the variant Trp354Leu destroyed one of the bonds with Glu324. The remaining variant (Pro249Leu) did not significantly affect hydrogen bonding but predicted to increased interaction with negative nucleotide, which might affect protein spatial structure.

We identified and confirmed two heterozygous de novo variants of c.1061G>T/p.(Trp354Leu) and c.746C>T/p.(Pro249Leu) in the *ATP6V1A* gene (Figure 2) which have never been described before (PS2). The two variants are not referenced as single nucleotide polymorphisms (SNPs) and not found in the HGMD professional database (http://hgmd.cf.ac.uk/), ClinVar database (http://www.ncbi.nlm/. http://nih.gov/clinvar/), human polymorphisms 1000 Genomes database, ExAC browser database, and gnomAD database (https://gnomad.broadinstitute.org/) (PM2_Supporting). *ATP6V1A* c.1061G>T and c.746C>T mutations were predicted as disease-causing (PP3) by four computational programs of SIFT (http://sift.jcvi.org/www/SIFT_enst_submit.html), PolyPhen-2 (http://genetics.bwh.harvard.edu/pph2/), Mutation Taster (http://www.mutationtaster.org/), and REVEL. We investigated the conservation of the missense substitution affecting p.Trp354 and p.Pro249 and found both sites are located in the highly conserved portion of the ATP6V1A polypeptide (Figure 2). We mapped the mutation sites of human *ATP6V1A* onto the crystal structure with Swiss model (Figure 2). Trp354 formed five hydrogen bonds with Glu324, Ser350, Thr351, Ala357, and Leu358, while the variant Trp354Leu destroyed one of the bonds with Glu324. The remaining variant (Pro249Leu) did not significantly affect hydrogen bonding but predicted to increased interaction with negative nucleotide, which might affect protein spatial structure. The novel missense variant c.746C>T/p.(Pro249Leu) is located at the same location where a pathogenic variant with different amino acid change p.Pro249Arg has been reported before (PM5). The *ATP6V1A* gene has a low rate of benign missense variation which is a common mechanism of the disorder (PP2). According to the 2015 American College of Medical Genetics and Genomics (ACMG) guidelines, both mutations of c.1061G>T/p.(Trp354Leu) and c.746C>T/p.(Pro249Leu) were all rated as likely pathogenic (evidence including PS2 + PM2_Supporting+PP2 + PP3 and PS2 + PM2_Supporting+PM5 + PP2 + PP3, individually).

### 3.3. Literature Review

We performed an exhaustive search of the literature using the PubMed and Chinese journals database (Wanfang Data) to compile clinical data on individuals with DEE93 (Table S1) using the following keywords: “ATP6V1A, DEE and Developmental and epileptic encephalopathy” or “DEE93.” Thirty-one patients with *ATP6V1A* pathogenic variants have been described in five articles (including 22 boys and nine girls), of whom 29 were sporadic and two were monozygotic twins, and all reported pathogenic variants were missense mutations. Despite harboring different variants, all patients demonstrated parallel neurological features as seizures (28/33), global developmental delay (29/33), and hypotonia in infancy (24/33). Seizures occurred in 23 patients within the first 3 years of age, and the youngest presented with seizures at 2 months old. The seizure types of patients associated with *ATP6V1A* variants included febrile seizures (10/28), infantile spasms (9/28), tonic seizures (11/28), clonic seizures (6/28), focal occipital seizures (6/28), generalized tonic-clonic seizures (7/28), and myoclonic jerks (6/28). Diffuse slowed background and multifocal epileptic discharge (13/26) were the most common types of EEG. Others include hypsarrhythmia (4/26), left central spike and slow waves (2/26), irregular slow rhythm in the occipital area during sleep (1/26), and temporal and frontal epileptiform discharges (1/26). Brain MRI of 22 patients showed characteristic findings that included hypomyelination in 13 patients, mild brain and cerebellar atrophy in 13 patients, thin corpus callosum in 4 patients, and bilateral lateral ventricle body broaden in one patient. Overall, language development impairment was a significant feature in DEE93 patients and correlated with the frequency and severity of seizures, with two-thirds of patients having no speech ability and one-third having poor language skills.

Congenital and secondary microcephaly was reported in half of individuals with *ATP6V1A* mutation. Two patients were described to have mild characteristic facial features including wide forehead, deep set eyes, and beaked nose. Other clinical features include optic atrophy, autistic traits, wide based gait, nonverbal, no visual fixation, coloboma of the iris, hypotonic dyskinetic quadriparesis eruption defects, defective enamel mineralization, excessive caries, and crumbling teeth. Comparison of our cases with previously reported cases associated with *ATP6V1A* mutation was summarized in Table S1.

A total of 31 pathogenic missense variants of *ATP6V1A* have been described in the literature, of which 21 variants are related to epileptic encephalopathy, 5 variants are found in patients with cutis laxa carrying *ATP6V1A* homozygous or compound heterozygous mutations, and the remaining 5 variants are from the cohort study of autism and encephalopathy. All of the pathogenic variants were missense (except for two nonsense mutations). Mapping the different pathogenic sites on the ATP6V1A genomic structure showed that accumulations were found in specific regions of the central *α*/*β* domain.

## 4. Discussion

The concept of DEE suggests that seizure activity may damage brain cells and lead to developmental delay and adverse neurodevelopmental consequences including cognitive retardation and intellectual disability. Under normal circumstances, melioration of the epileptiform activity may ameliorate the developmental consequences of these disorders [19, 20]. Studies showed that epileptic encephalopathy caused by genetic mutations may disrupt normal brain function and subsequently lead to the development of encephalopathy and epilepsy [2]. Hence, in some severe genetic epilepsy in infancy and childhood, the effects on intelligence, cognition, and development may be due to the direct effect of genetic mutations, rather than the developmental effect of frequent epileptic activity.

*ATP6V1A* mutation in patient with DEE93 is a rare neurologic disorder characterized by developmental delay, early-onset refractory seizures, hypotonia, and intellectual disability. Our review of reported patients with *ATP6V1A* dominant mutations shows that epilepsy is usually the first symptom. How to choose the appropriate combination of antiepileptic drugs is the primary problem that needs to be solved. Kadwa reported an Indian boy with infantile spasms who was treated with ACTH, valproic acid, levetiracetam, and zonisamide sequentially [17]. Antiepileptic drug treatment effectively decreased the frequency of seizures, but the intellectual disability and developmental delay caused by genetic defects remain profound. Studies showed that *ATP6V1A* dominant variants in patients caused a similar defect in neurite elongation accompanied by loss of excitatory inputs and impaired synapse formation, revealing that altered lysosomal homeostasis markedly affects neurite development and synaptic connectivity which leads to intellectual disability, developmental delay, and epilepsy [9]. The role of V-ATPase in pH homeostasis and intracellular signalling pathways is ubiquitous, but it is highly significant in neurons where it plays additional role in neurotransmitter loading into synaptic vesicles and synaptic transmission [21, 22]. So the antiseizure medications modulating the release of neurotransmitters through presynaptic action (e.g., levetiracetam, brivaracetam, gabapentin, and pregabalin) may be effective. Controlling the frequency of seizures has positive implications for treatment in epileptic encephalopathy patients, but effective control of seizures does not mean that developmental delays and intellectual disabilities are improved.

In the present study, we identified two heterozygous *ATP6V1A* variants in two unrelated cases. Initially, Patient 1 had a cluster of four to five seizures a day, accompanied by nodding and hugging movements. We tried a treatment option with levetiracetam, vigabatrin, and ketogenic diet for 6 months. Excitingly, her frequency of the seizures was effectively reduced, and repeat interictal EEG was also improved. On follow-up after 3 months of the treatment, her seizures did not subside completely, while the seizures frequency tended to be stable. Patient 2 had development delay after birth, and seizures were not detected until 1 year and 5 months of age. ATP6V0A2-related cutis laxa seizures often appear later in the course of the disease in patients that initially only show a mild developmental delay with speech delay [23]. This seems to be a recurrent theme in the V-ATPase-related epilepsies. Therefore, although Patient 2 currently only shows developmental delay without seizures, she is still at risk of developing epilepsy during development and needs to be closely monitored during her future care.

Functional studies have shown that changes in the V-ATPase catalytic function or stability form the basis of the abnormal phenotypes. In severely affected patients, they exhibited reduced expression of the glycoprotein LAMP1 and increased endo-lysosomal pH, suggesting that reduced proton pump activity may result in loss of ATP6V1A function or loss of expression due to protein degradation. In mild–moderate affected patients, there is no alteration of the glycoprotein LAMP1 expression and endo-lysosomal pH reduction, suggesting a gain in ATP6V1A function [10, 11, 18]. Therefore, the severity of the clinical phenotype in DEE93 patients is related to the expression level of the glycoprotein LAMP1 and the alteration of endo-lysosomal pH. Based on above evidence, we speculate that severely affected patient carrying p.Trp354Leu variant was potentially associated with the LOF of ATP6V1A, and slightly affected patient carrying p.Pro249Arg variant was potentially associated with the GOF of ATP6V1A, which requires further functional studies.

In conclusion, we herein report two de novo pathogenic missense mutations in two patients presenting syndromic developmental delay with or without epilepsy. We emphasized on the clinical phenotypic variability and genotypic characteristics, which will hopefully lead to more individuals to be diagnosed and help expand our understanding of DEE. Meanwhile, in order to further investigate the potential genotype–phenotype correlations of *ATP6V1A* mutations with DEE, more detailed clinical case reports, including information of *ATP6V1A* mutations and their clinical consequences, will be needed.

## Data Availability

The data that support the findings of this study are available from the corresponding author Yi Liu (y_liu99@sina.com) upon reasonable request.

## Supporting Information

Supporting Information Additional supporting information can be found online in the Supporting Information section. Table S1: review of all cases genetic and clinical features with the developmental and epileptic encephalopathy 93.
